# Single-cell analysis reveals KLF10-high macrophages induced by glioblastoma cells are associated with tumor progression

**DOI:** 10.3389/fimmu.2026.1820802

**Published:** 2026-07-20

**Authors:** Xiaodong Zhang, Caiping Chen, Qianya Fan, Sheng Chen, Duanzheng Cao, Zhonglin Hu, Yejun Zhou, Jinfang Xu, Delin Wang

**Affiliations:** 1 Department of Neurosurgery, Jiande First People’s Hospital, Hangzhou, Zhejiang, China; 2 Jiande Blood Bank Center, Hangzhou, Zhejiang, China; 3 Department of Neurology, Jiande First People’s Hospital, Hangzhou, Zhejiang, China; 4 Department of Neurosurgery, The Second Affiliated Hospital Zhejiang University School of Medicine, Hangzhou, Zhejiang, China

**Keywords:** KLF10, recurrent glioblastoma, single-cell RNA sequencing, tumor microenvironment, tumor-associated macrophages

## Abstract

**Background:**

Glioblastoma (GBM) is the most common and aggressive primary malignant tumor of the adult central nervous system, with nearly 90% of cases recurring within two years despite standard surgery, radiotherapy, and chemotherapy. Tumor-associated macrophages (TAMs) play critical roles in GBM recurrence, but their characteristics in recurrent GBM remain insufficiently defined. Krüppel-like factor 10 (KLF10) regulates metabolism, mitochondrial function, proliferation, and apoptosis; however, its role in GBM-associated TAMs remains unclear.

**Methods:**

Single-cell RNA-sequencing data from 16 GBM samples were analyzed to identify recurrence-associated TAMs. Functional features were assessed using GO, KEGG, and CellChat analyses. Marker genes were used to construct a prognostic signature through LASSO and Cox regression. The association between KLF10 and clinicopathological features was evaluated using TCGA data. A GBM cell–macrophage co-culture system was established to examine KLF10 induction, and KLF10-overexpressing macrophages were used to assess effects on GBM-cell proliferation, migration, and invasion.

**Results:**

A five-gene prognostic signature comprising KLF10, ZFAND2A, SMAD7, GPR84, and ARHGAP12 was identified. KLF10 expression was associated with WHO grade, IDH mutation, MGMT promoter methylation, 1p/19q co-deletion, molecular subtype, and overall survival, with high KLF10 predicting poor prognosis. Functional enrichment analyses suggested that KLF10-associated genes were linked to ECM–receptor interaction, cell-cycle-related pathways, and cytokine signaling. *In vitro*, co-culture with GBM cells upregulated KLF10 expression in THP-1-derived macrophages, and KLF10-overexpressing macrophages were associated with increased GBM-cell proliferation, migration, and invasion.

**Conclusions:**

KLF10-high macrophages were associated with GBM progression and may serve as a candidate prognostic marker requiring further validation.

## 1 Introduction

As the most common and lethal primary malignancy of the adult central nervous system, glioblastoma constitutes over half of all malignant CNS tumors, with an annual incidence of approximately 4.03 cases per 100, 000 population (1). Despite advances in multimodal therapy that include maximal surgical debulking, temozolomide-based chemoradiotherapy, and postoperative adjuvant treatment, the clinical outcome of glioblastoma remains extremely poor, 90% of glioblastomas relapsed within two years of diagnosis (2). The pronounced invasiveness, rapid proliferative capacity, and almost inevitable recurrence of this tumor collectively contribute to a median survival time of less than 15 months (3). Therefore, a deeper understanding of the cellular composition, functional states, and regulatory mechanisms of the glioblastoma immune microenvironment is essential for the identification of molecules associated with tumor progression and the development of more effective therapeutic strategies.

TAMs are one of the most prominent infiltrating immune cell types in the glioblastoma tumor microenvironment and exhibit significant heterogeneity. TAMs include Mo-TAMs derived from circulating monocytes and Mg-TAMs originating from resident microglia, both of which play critical roles in tumor progression (4). Compared to primary glioblastoma, recurrent glioblastoma differs in molecular and histological features, tumor heterogeneity, and immune microenvironment, all of which contribute to the invasiveness and treatment resistance of rGBM (5). Previous studies have found that TAMs were significantly infiltrated in recurrent glioblastoma after anti-angiogenic therapy, and may promote tumor progression and resistance through immune evasion mechanisms (6). In recurrent glioblastoma (rGBM), the tumor microenvironment is characterized by abundant immunosuppressive TAMs, which restrict the efficacy of immunotherapy (7). However, the mechanisms by which tumor cells in rGBM reprogrammed macrophages, and how these macrophages may contribute to tumor progression, remained poorly understood.

Krüppel−like factors (KLFs) constitute a highly conserved subfamily of zinc−finger transcription factors in eukaryotes, comprising 17 members (KLF1–KLF17) characterized by three classical C2H2 zinc−finger domains at the C−terminus that bind GC/GT−rich DNA elements to regulate downstream gene expression (8). Kruppel-like factor 10 (KLF10), also known as the transforming growth factor beta-inducible early gene 1 (TIEG1), was first identified in human osteoblasts (9). In recent years, studies have found that KLF10 influenced tumor progression in various types of cancer (10, 11). However, the role of KLF10 in glioblastoma, particularly in the glioblastoma immune microenvironment, remained unclear.

## 2 Materials and methods

### 2.1 Single-cell and bulk RNA-seq datasets and preprocessing

Single-cell RNA-sequencing (scRNA-seq) data derived from 16 glioblastoma specimens were retrieved from the GSE182109 dataset. The primary or recurrent status of each sample was determined according to the clinical/sample metadata provided in the original dataset, in which samples had been annotated as primary GBM or recurrent GBM. Data filtering, normalization, clustering, and downstream analyses were carried out using the Seurat R package (v4.0). To minimize inter-sample batch effects, scRNA-seq data from different GBM specimens were integrated using the Seurat integration workflow and further corrected with Harmony according to the original sample identity before clustering and UMAP visualization. Cells with fewer than 200 detected genes or with mitochondrial gene proportions exceeding 20% were removed. Dimensionality reduction was first performed by principal component analysis (PCA), followed by Uniform Manifold Approximation and Projection (UMAP) for low-dimensional visualization.

Bulk RNA-seq data of glioblastoma (GBM), lower-grade glioma (LGG), and normal brain tissues were collected from publicly available TCGA and GTEx resources using the UCSC Xena data portal (https://xenabrowser.net/datapages/). The Mo-TAM2 score was calculated using the GSVA method based on the marker genes identified from the Mo-TAM2 subcluster. The Mo-TAM2 marker gene set used for GSVA scoring is listed in **Supplementary Table 1**.

### 2.2 Cell–cell communication analysis

Cell–cell communication was analyzed using the CellChat R package (version 2.1.2). Based on normalized single-cell expression data and cell-type annotations, ligand–receptor interactions were inferred using the built-in human CellChat database. Communication probabilities were calculated to identify significant signaling interactions between cell populations, and signaling networks were summarized and visualized using default CellChat workflows.

### 2.3 Functional enrichment analysis

Functional enrichment analyses were performed to interpret the biological roles of differentially expressed genes (DEGs). Gene Ontology (GO) terms and Kyoto Encyclopedia of Genes and Genomes (KEGG) pathways were assessed using the clusterProfiler R package. Enrichment significance was evaluated after multiple-testing correction, and terms with an adjusted P value < 0.05 were considered statistically significant.

### 2.4 LASSO and Cox regression analysis

To identify prognostic genes associated with overall survival, least absolute shrinkage and selection operator (LASSO) Cox regression analysis was performed using the glmnet R package. Gene expression data were incorporated into a Cox proportional hazards model, and the optimal penalty parameter (λ) was determined by 10-fold cross-validation. Genes with non-zero coefficients were retained to construct a prognostic risk score model. The predictive performance of the model was subsequently evaluated using Cox regression analysis.

### 2.5 Cell culture and transfection

Human glioblastoma cell lines SNB19 and LN229 were obtained from the American Type Culture Collection (ATCC, USA) and maintained in Dulbecco’s Modified Eagle Medium (DMEM; Gibco, USA) supplemented with 10% fetal bovine serum (FBS; ScienCell, USA). Cells were cultured at 37 °C in a humidified atmosphere containing 5% CO_2_. The human monocytic cell line THP-1 (ATCC, USA) was cultured in RPMI-1640 medium (Gibco, USA) containing 10% heat-inactivated FBS. To induce macrophage differentiation, THP-1 cells were seeded at a density of 1 × 10^6^ cells/mL and stimulated with 100 ng/mL phorbol 12-myristate 13-acetate (PMA; MCE, USA) for 48 h.

### 2.6 Plasmid overexpression

Gene overexpression plasmids and the corresponding empty vector controls were constructed and obtained from GenePharma (Shanghai, China). Transfections were carried out using Lipofectamine 3000 (Invitrogen, USA) in accordance with the manufacturer’s instructions. Cells were harvested 48 h after transfection for subsequent analyses.

### 2.7 RNA isolation and quantitative real-time PCR

Total RNA from cultured cells or tissue specimens was isolated using TRIzol reagent (Invitrogen, USA) following the supplier’s guidelines. Reverse transcription for mRNA analysis was carried out with the All-in-One Ultra RT SuperMix (Vazyme, China). Quantitative real-time PCR was performed using ChamQ Blue Universal SYBR qPCR Master Mix (Vazyme, China) on an ABI 7500 Real-Time PCR system (Applied Biosystems, USA). GAPDH was used as the endogenous control for normalization. Relative expression was determined using the 2^−ΔΔCt approach. Each sample was assayed in technical triplicates. The primer sequences used for quantitative real-time PCR were as follows: KLF10, forward: 5′- CTTCCGGGAACACCTGATTTT-3′, reverse: 5′- GCAATGTGAGGTTTGGCAGTATC-3′; GAPDH, forward: 5′- CAGGAGGCATTGCTGATGAT-3′, reverse: 5′-GAAGGCTGGGGCTCATTT-3′.

### 2.8 Transwell migration and invasion assays

Cell migration and invasion were examined using Transwell chambers with 8.0 μm pore polycarbonate membranes (Corning, USA). For migration assays, 5 × 10^4^ glioblastoma cells suspended in 200 μL medium were seeded into the upper chamber, while 5 × 10^4^ macrophages in 600 μL medium were added to the lower chamber as a chemoattractant. After 48 h of incubation at 37 °C with 5% CO_2_, non-migrated cells were removed, and migrated cells were fixed with 4% paraformaldehyde, stained with 1% crystal violet, and counted under a light microscope.

For invasion assays, the upper chambers were precoated with Matrigel (1:8 dilution; Corning, USA), and the remaining procedures were performed as described for the migration assay.

### 2.9 Cell proliferation assay

Cell viability was evaluated using the Cell Counting Kit-8 assay (CCK-8; Dojindo, Japan). Glioblastoma cells were plated in 96-well plates at a density of 3 × 10^3^ cells per well in 100 μL of complete DMEM. Wells containing medium and CCK-8 reagent without cells were used as background controls. At the indicated time intervals (every 24 h), 10 μL of CCK-8 reagent was added to each well, followed by incubation at 37 °C for 2 h. Absorbance was measured at 450 nm using a microplate reader (Tecan, Switzerland). Each experiment was conducted in triplicate.

### 2.10 Flow cytometric analysis

Cells were collected, washed with PBS, and stained with a fluorophore-conjugated anti-CD11b antibody (BioLegend, USA) for 20 min at room temperature in the dark. Fc receptor blocking was not performed in this specific flow cytometry assay, as CD11b staining was used only to confirm PMA-induced THP-1 macrophage-like differentiation. After washing, samples were analyzed on a Navios flow cytometer (Beckman Coulter, USA), and data were processed using FlowJo software. The CD11b-positive gate was defined using unstimulated THP-1 cells as the negative control. The gating strategy is shown in **Supplementary Figure 1**.

### 2.11 Wound healing assay

Cell migratory ability was evaluated using a wound healing assay. Transfected cells were seeded into culture plates and grown to near confluence. A straight scratch was generated using a sterile pipette tip, and detached cells were gently removed by washing with PBS. Cells were then maintained in serum-free medium, and images of the wound area were captured at 0 and 24 h under a light microscope. Wound closure was quantified by comparing the remaining wound width at each time point. All experiments were performed in triplicate.

### 2.12 EdU incorporation assay

Cell proliferation was evaluated using a 5-ethynyl-2′-deoxyuridine (EdU) incorporation assay with a commercial EdU detection kit (Beyotime Biotechnology, China), following the manufacturer’s instructions. Cells were seeded onto coverslips and incubated with EdU reagent for the indicated duration. After labeling, cells were fixed with 4% paraformaldehyde, permeabilized with 0.5% Triton X-100, and subjected to the Click reaction to detect EdU incorporation. Nuclei were counterstained with Hoechst, and images were captured using a fluorescence microscope. The proportion of EdU-positive cells was calculated from randomly selected fields. All experiments were performed in triplicate.

### 2.13 Statistical analysis

Statistical analyses were conducted using GraphPad Prism software (version 9.3; GraphPad Software, USA) and R software where applicable. Quantitative experimental data are presented as mean ± standard deviation (SD) from at least three independent experiments. For comparisons between two groups, a two-tailed unpaired Student’s t-test or Mann–Whitney U test was used according to data distribution. For comparisons among multiple groups, one-way ANOVA or Kruskal–Wallis test was used where applicable.

For transcriptomic and enrichment analyses, multiple-testing correction was applied where appropriate, and adjusted P values were used to determine statistical significance. GO and KEGG enrichment analyses were performed using the clusterProfiler R package, with an adjusted P value < 0.05 considered statistically significant.

The prognostic model was constructed using LASSO Cox regression with 10-fold cross-validation to determine the optimal penalty parameter λ. To improve gene-selection stability, 100 repeated LASSO selections were performed, and genes with a selection frequency greater than 75 were retained for model construction. Survival differences were evaluated using Kaplan–Meier analysis and the log-rank test. Cox regression analysis was used to estimate hazard ratios (HRs) and 95% confidence intervals (CIs) where applicable. Time-dependent ROC curves, calibration curves, and decision curve analysis were used to evaluate the predictive performance and potential clinical utility of the prognostic model. A P value < 0.05 was considered statistically significant unless otherwise specified. For experimental data, ns, not significant; *P < 0.05; **P < 0.01; ***P < 0.001.

## 3 Results

### 3.1 Identification of marker gene expression profiles of macrophages associated with glioblastoma recurrence

**Figure 1:**
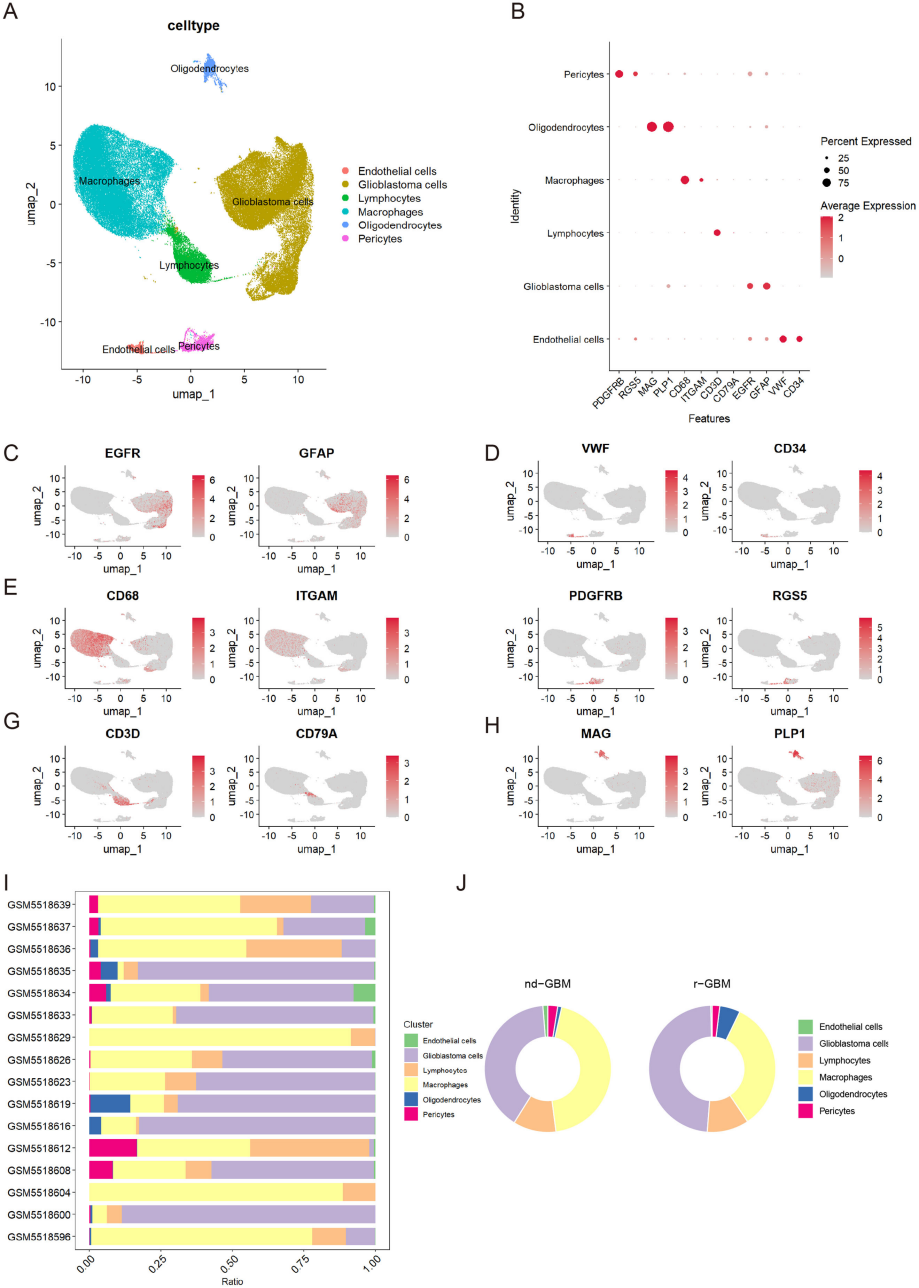
Cellular composition of primary and recurrent glioblastoma revealed by single-cell RNA sequencing **(A)** UMAP visualization showing six major cell populations. **(B)** Dot plot showing representative marker gene expression across major cell types. **(C–H)** Feature plots showing the expression of canonical marker genes used for cell-type annotation. **(I)** Proportional distribution of major cell populations across individual glioblastoma samples, demonstrating substantial inter-sample heterogeneity in cellular composition. **(J)** Comparison of cell-type proportions between primary and recurrent glioblastoma samples, showing that glioblastoma cells and macrophages constituted the dominant cell populations in both tumor states.

**Figure 2:**
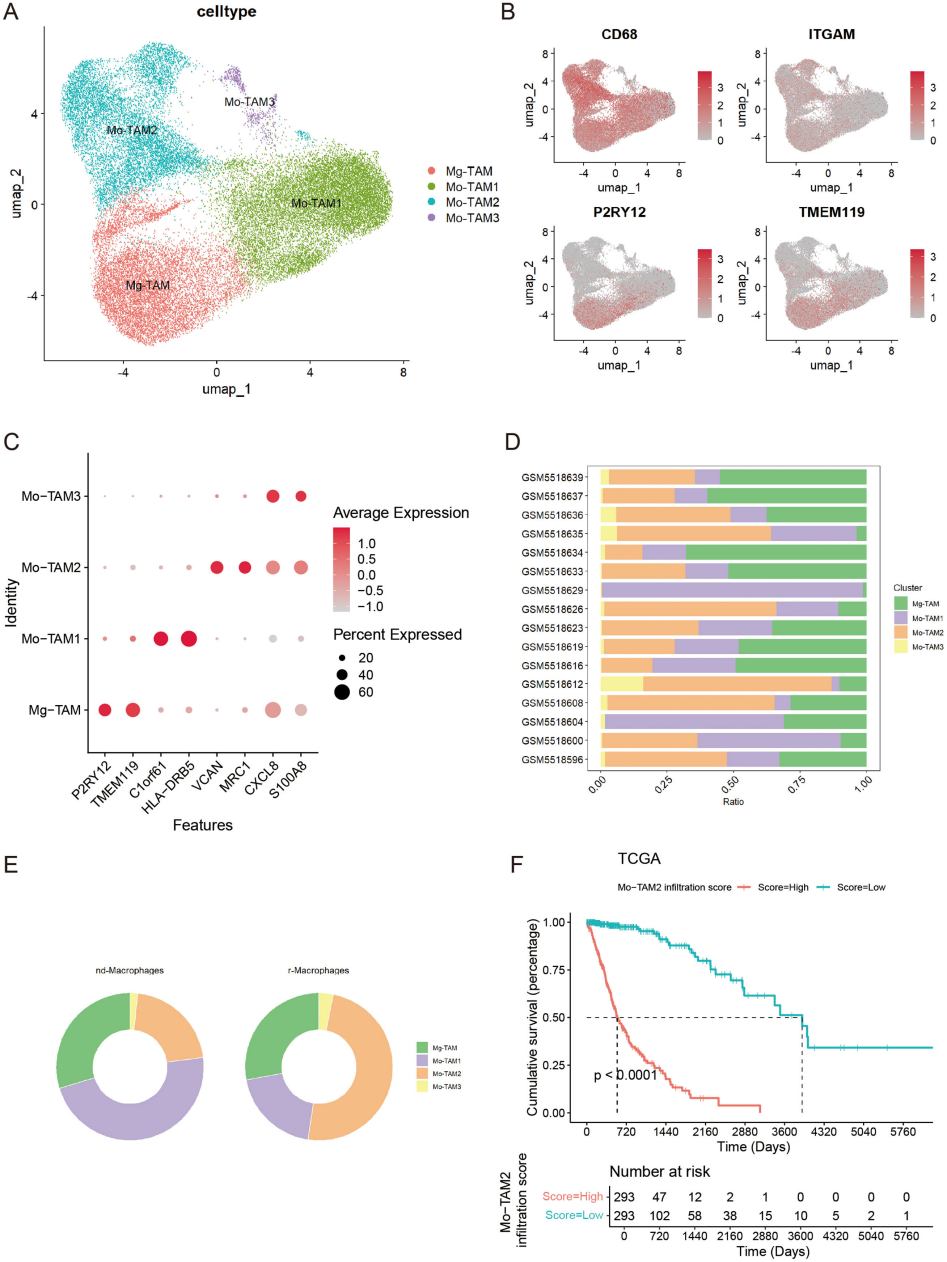
Heterogeneity of tumor-associated macrophage subpopulations in primary and recurrent glioblastoma **(A)** UMAP plot showing four distinct TAM subpopulations, including three monocyte-derived TAM clusters (Mo-TAM1, Mo-TAM2, and Mo-TAM3) and one microglia-derived TAM cluster (Mg-TAM). **(B)** Marker-based annotation of TAM subpopulations. **(C)** Dot plot showing the expression of representative marker genes across TAM subclusters. **(D)** Relative proportions of TAM subpopulations across individual glioblastoma samples, indicating marked inter-sample variability. **(E)** Comparison of TAM subpopulation proportions between primary and recurrent glioblastoma, showing a significant decrease in Mo-TAM1 and a marked increase in Mo-TAM2 in recurrent tumors, while Mg-TAM and Mo-TAM3 proportions remained largely unchanged. **(F)** Kaplan–Meier survival analysis demonstrating that higher infiltration of Mo-TAM2 was significantly associated with poorer overall survival in patients with glioblastoma.

The scRNA-seq data from 16 glioblastoma samples, both primary and recurrent, were extracted from the GSE182109 dataset. Data preprocessing, including quality control and clustering, was carried out using Seurat (v4.0) in R. Dimensionality reduction was performed using principal component analysis (PCA), followed by visualization through uniform manifold approximation and projection (UMAP). To evaluate whether the observed clusters were driven by patient-specific batch effects, we visualized the UMAP distribution according to original sample identity. Cells from different GBM samples were broadly intermixed across the major cell populations, indicating that the clusters were not dominated by individual patients and supporting the effectiveness of batch-effect correction (**Supplementary Figure 2A**). Six distinct cell populations were identified: Glioblastoma cells, Macrophages, Lymphocytes, Oligodendrocytes, Pericytes, and Endothelial cells (**Figure 1A**). To further confirm the neoplastic identity of the GBM cell population, we performed CNV inference using inferCNV. Compared with normal/reference cell populations, the annotated GBM cells exhibited broad chromosomal copy number alterations, supporting their malignant identity (**Supplementary Figure 2B**). The identities of these major cell populations were annotated based on canonical marker gene expression. Glioblastoma cells were characterized by the expression of EGFR and GFAP; macrophages were identified by CD68 and ITGAM; lymphocytes were annotated according to CD3D and CD79A expression; endothelial cells were marked by VWF and CD34; oligodendrocytes were defined by MAG and PLP1; and pericytes were identified by PDGFRB and RGS5 (**Figures 1B–H**). These marker expression patterns supported the reliability of the major cell-type annotations. There were significant differences in the proportions of cell populations across different glioblastoma samples, with a clear variation in the distribution of cell populations between samples. However, the majority of the samples were predominantly composed of glioblastoma cells and macrophages (**Figure 1I**). Glioblastoma cells and macrophages were the predominant cell types in primary and recurrent glioblastoma (**Figure 1J**). To further investigate the macrophage subpopulation characteristics in glioblastoma, macrophages were extracted and reclustered into four distinct subpopulations: three monocyte-derived tumor-associated macrophages (Mo-TAMs) clusters and one microglia-derived tumor-associated macrophages (Mg-TAMs) cluster (**Figures 2A, B**). The dot plot displayed representative marker genes for each TAM subcluster (**Figure 2C**). Significant differences were observed in the proportions of TAM subpopulations among different samples (**Figure 2D**). Compared with primary glioblastoma, the proportion of Mo-TAM1 was markedly decreased in recurrent glioblastoma, whereas the proportion of Mo-TAM2 was significantly increased; the proportions of Mg-TAM and Mo-TAM3 did not show significant changes (**Figure 2E**). The association between Mo-TAM2 characteristics and clinical prognosis in glioblastoma was evaluated using Kaplan–Meier analysis. Patients with glioblastoma exhibiting higher Mo-TAM2 cell infiltration showed significantly poorer survival outcomes (**Figure 2F**).

### 3.2 GO and KEGG enrichment analysis of Mo-TAM2

**Figure 3:**
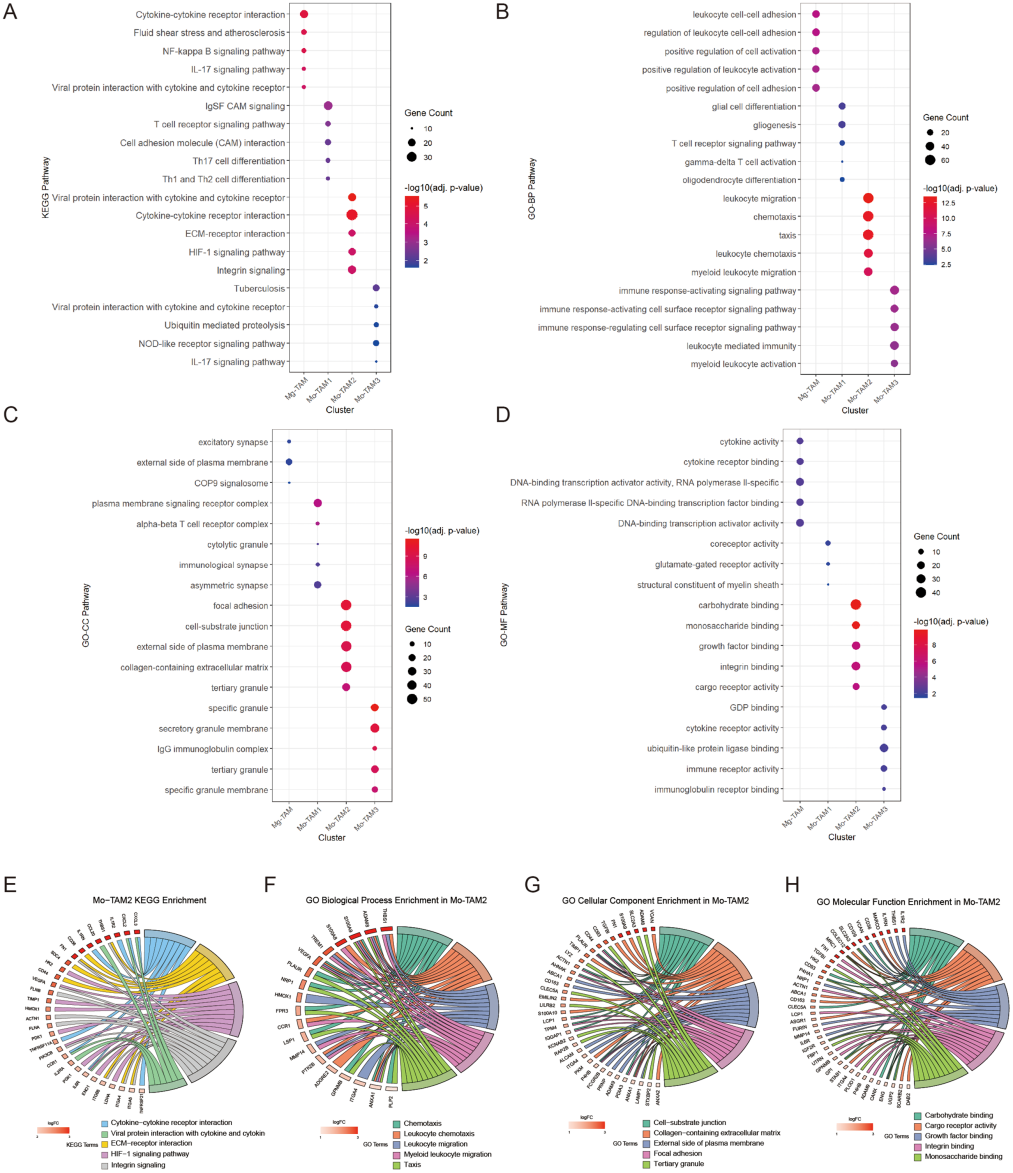
GO and KEGG enrichment analyses of the Mo-TAM2 subpopulation **(A)** KEGG pathway enrichment analysis revealed that Mo-TAM2 was significantly enriched in pathways associated with inflammation and tumor microenvironment remodeling, including cytokine–cytokine receptor interaction, ECM–receptor interaction, HIF-1 signaling pathway, integrin signaling, and viral protein interaction with cytokine and cytokine receptor. **(B)** GO biological process analysis demonstrated that Mo-TAM2-associated genes were predominantly involved in cell motility and immune trafficking programs, such as leukocyte migration, leukocyte chemotaxis, chemotaxis, taxis, and myeloid leukocyte migration. **(C)** GO cellular component enrichment indicated that Mo-TAM2-related genes were mainly localized to focal adhesion, cell–substrate junction, the external side of the plasma membrane, and collagen-containing extracellular matrix. **(D)** GO molecular function analysis showed significant enrichment in integrin binding, growth factor binding, carbohydrate binding, monosaccharide binding, and cargo receptor activity, suggesting active participation in ligand–receptor interactions and extracellular signaling. **(E)** Chord diagram illustrating the associations between representative genes and enriched KEGG pathways in Mo-TAM2. **(F–H)** Chord diagrams depicting the relationships between representative genes and enriched GO biological processes, cellular components, and molecular functions.

KEGG pathway enrichment analysis showed that Mo-TAM2 was significantly enriched in pathways related to inflammation and tumor microenvironment remodeling, including cytokine–cytokine receptor interaction, ECM–receptor interaction, HIF-1 signaling pathway, and integrin signaling. In addition, Mo-TAM2 also exhibited enrichment in immune-related pathways such as viral protein interaction with cytokine and cytokine receptor (**Figure 3A**).

GO biological process analysis revealed that Mo-TAM2 was predominantly associated with cell motility and immune trafficking programs, including leukocyte migration, leukocyte chemotaxis, chemotaxis, taxis, and myeloid leukocyte migration, indicating an enhanced migratory and recruitment capacity (**Figure 3B**). Consistently, GO cellular component enrichment demonstrated that Mo-TAM2-associated genes were mainly localized to focal adhesion, cell–substrate junction, external side of plasma membrane, and collagen-containing extracellular matrix, supporting a role in cell–matrix interaction and extracellular remodeling (**Figure 3C**). GO molecular function analysis further showed that Mo-TAM2 was enriched in integrin binding, growth factor binding, carbohydrate binding, monosaccharide binding, and cargo receptor activity, suggesting functional involvement in ligand–receptor interactions and extracellular signaling (**Figure 3D**).

Chord diagrams illustrated the relationships between representative genes and enriched KEGG pathways (**Figure 3E**), as well as GO biological processes (**Figure 3F**), cellular components (**Figure 3G**), and molecular functions (**Figure 3H**), highlighting the central role of Mo-TAM2 in immune regulation, extracellular matrix interaction, and inflammatory signaling within the glioblastoma microenvironment.

### 3.3 Cell–cell communication between Mo-TAM2 and glioblastoma cells

**Figure 4:**
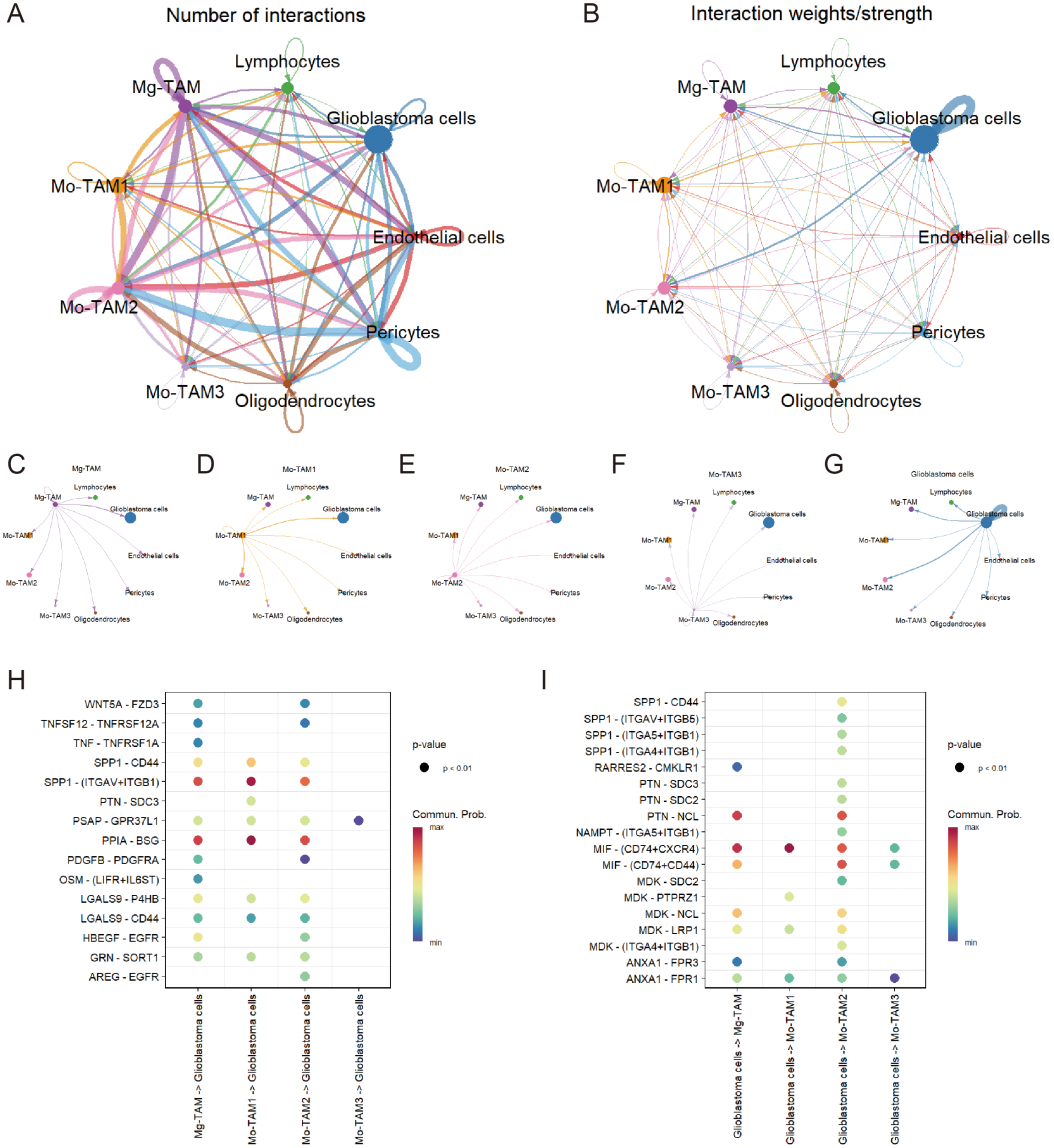
Cell–cell communication between Mo-TAM2 and glioblastoma cells **(A, B)** Global interaction networks based on the number of ligand–receptor pairs showing extensive communication between TAM subsets and multiple cell populations, including glioblastoma cells, endothelial cells, pericytes, oligodendrocytes, and lymphocytes. **(C–G)** Quantitative analysis of interaction strength revealed that Mo-TAM2 exhibited relatively strong and frequent communication signals with glioblastoma cells compared with other TAM subpopulations. **(H, I)** Ligand–receptor interaction analysis highlighting representative signaling axes mediating Mo-TAM2–glioblastoma cell communication.

Cell–cell communication analysis was performed to characterize the interaction patterns between TAM subpopulations and other cell types in glioblastoma. The global interaction network based on the number of ligand–receptor interactions revealed extensive communication between TAM subsets and multiple cell populations, including glioblastoma cells, endothelial cells, pericytes, oligodendrocytes, and lymphocytes (**Figures 4A, B**). Among TAM subsets, Mo-TAM2 exhibited frequent interactions with glioblastoma cells and stromal components. Analysis of interaction strength further demonstrated that Mo-TAM2 displayed relatively strong communication signals with glioblastoma cells (**Figures 4C–G**).

At the ligand–receptor level, Mo-TAM2–associated communication with glioblastoma cells was characterized by multiple signaling axes, including SPP1–CD44, AREG-EGFR, PTN-SDC2/3 and so on (**Figures 4H, I**).

Collectively, these results suggested that Mo-TAM2 exhibited enhanced and specific cell–cell communication with glioblastoma cells, particularly through cytokine, adhesion, and extracellular matrix associated signaling pathways, highlighting its potential role in shaping the glioblastoma microenvironment.

### 3.4 Construction of a Mo-TAM2–based prognostic signature in glioblastoma

**Figure 5:**
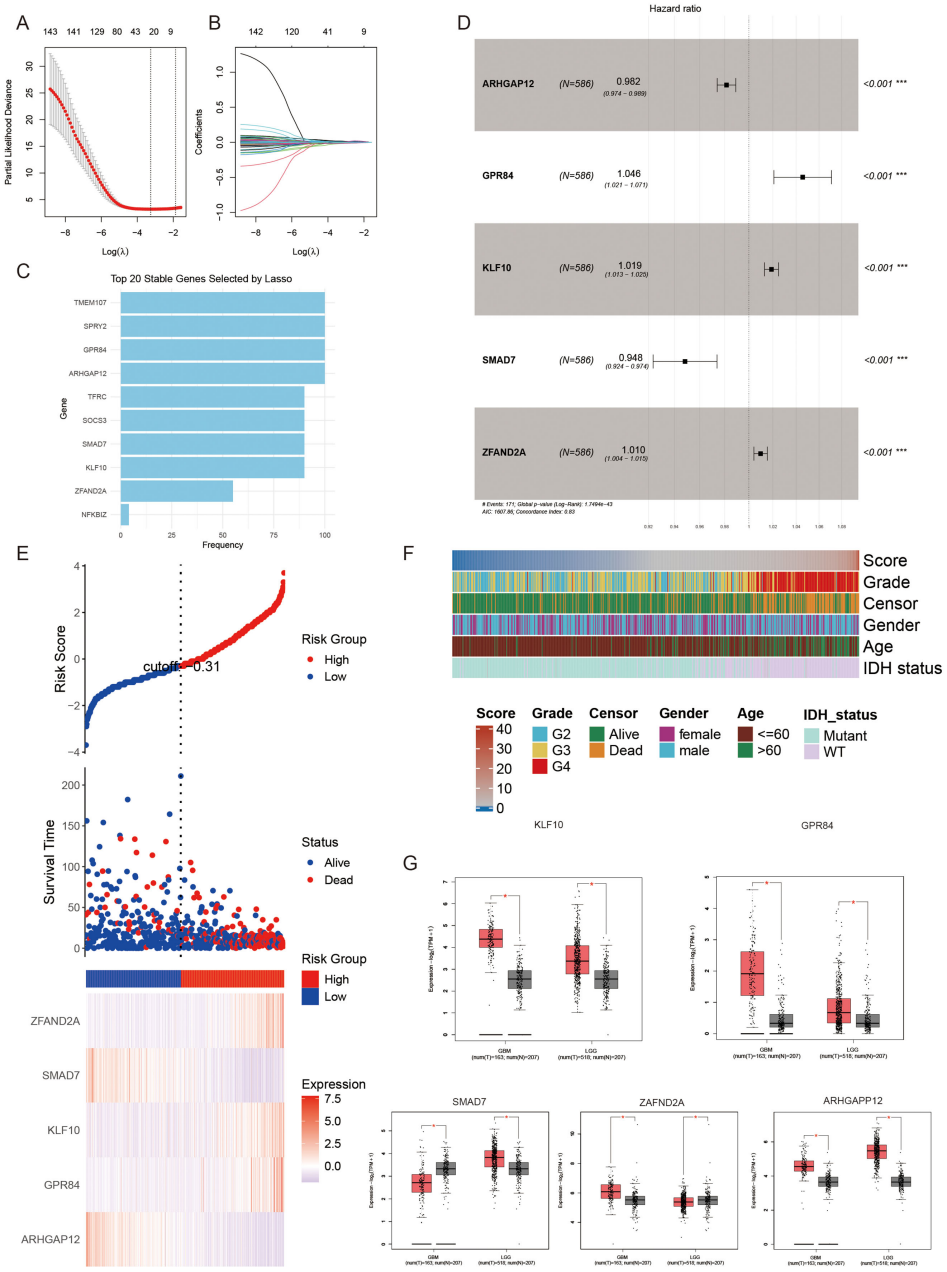
Construction of a Mo-TAM2–based prognostic signature in glioblastoma **(A)** Cross-validation plot showing selection of the optimal penalty parameter (λ) based on the minimum partial likelihood deviance. **(B)** Coefficient profiles of candidate genes across different λ values during LASSO regression. **(C)** Gene selection frequency after 100 repeated LASSO iterations, with genes selected more than 75 times considered stable and retained for model construction. **(D)** Multivariate Cox regression analysis demonstrating that the selected genes independently contributed to overall survival. **(E)** Distribution of risk scores and corresponding survival status of patients stratified into high- and low-risk groups based on the optimal cutoff value. **(F)** Heatmap illustrating the expression patterns of prognostic genes in high- and low-risk groups, together with clinical characteristics including tumor grade, survival status, gender, age, and IDH mutation status. **(G)** Differential expression analysis showing that SMAD7, ZFAND2A, ARHGAP12, KLF10, and GPR84 were significantly upregulated in glioblastoma and lower-grade glioma samples compared with normal brain tissues.

To evaluate the prognostic value of Mo-TAM2 marker genes, a prognostic risk model was constructed using LASSO Cox regression analysis. Cross-validation was performed to determine the optimal penalty parameter (λ) based on the minimum partial likelihood deviance (**Figure 5A**), and the coefficient trajectories of candidate genes across different λ values were visualized (**Figure 5B**). After 100 repeated LASSO selections, genes with a selection frequency greater than 75 were considered stable and retained for subsequent model construction (**Figure 5C**). Multivariate Cox regression analysis further demonstrated that the selected genes independently contributed to patient prognosis (**Figure 5D**).

A risk score was calculated for each patient according to the expression levels and corresponding coefficients of the selected genes. Patients were stratified into high and low risk groups based on the optimal cutoff value (**Figure 5E**). The distribution of risk scores and survival status indicated that patients in the high-risk group exhibited higher mortality and shorter survival time.

The heatmap illustrated the expression patterns of the prognostic genes across different risk groups, together with clinical characteristics including tumor grade, survival status, gender, age, and IDH mutation status (**Figure 5F**). Furthermore, differential expression analysis showed that SMAD7, ZFAND2A, ARHGAP12, KLF10, and GPR84 exhibited higher expression levels in glioblastoma and lower-grade glioma samples compared with normal tissues (**Figure 5G**).

**Figure 6:**
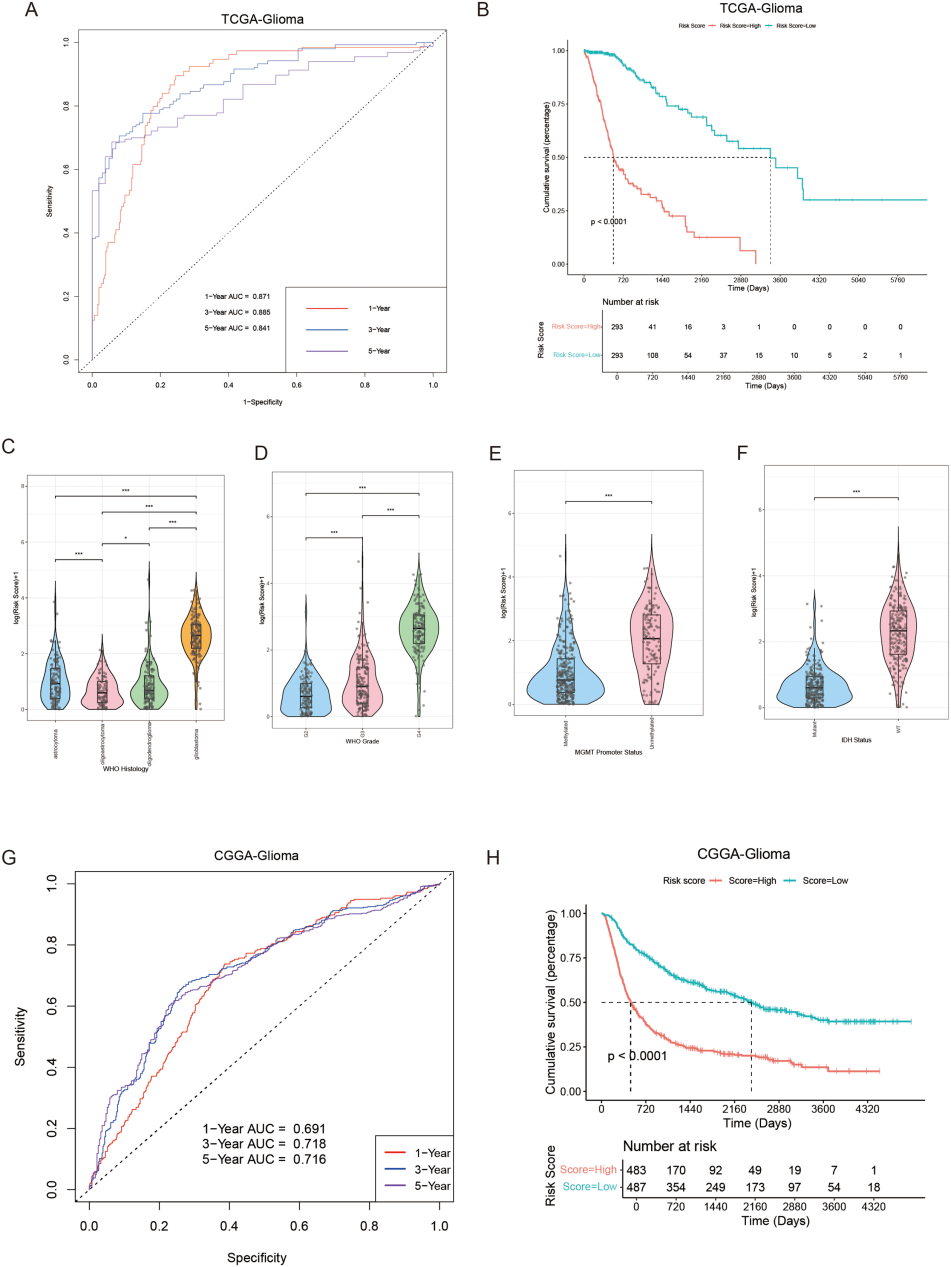
Prognostic performance and clinical relevance of the Mo-TAM2–based risk model **(A)** Time-dependent receiver operating characteristic (ROC) curves showing favorable predictive accuracy of the risk model for 1-, 3-, and 5-year overall survival. **(B)** Kaplan–Meier survival analysis demonstrating significantly shorter overall survival in the high-risk group compared with the low-risk group. **(C)** Comparison of risk scores among glioblastoma, lower-grade glioma, and normal brain tissues, showing significantly higher risk scores in glioblastoma samples. **(D)** Risk score distribution according to WHO grade, revealing a progressive increase in risk scores with advancing tumor grade. **(E)** Comparison of risk scores between patients with methylated and unmethylated MGMT promoter status. **(F)** Comparison of risk scores between IDH-mutant and IDH wild-type gliomas, showing higher risk scores in IDH wild-type tumors. **(G)** ROC analysis from the CGGA glioma cohort further validating the predictive accuracy and clinical relevance of the Mo-TAM2–based risk model. AUC values for 1-, 3-, and 5-year survival are 0.691, 0.718, and 0.716, respectively. **(H)** Kaplan–Meier survival analysis from the CGGA glioma cohort, demonstrating that patients in the high-risk group had significantly shorter survival compared with those in the low-risk group (p < 0.0001), confirming the model’s robustness across independent datasets.

The predictive performance of the Mo-TAM2–based prognostic model was further evaluated. Time-dependent receiver operating characteristic (ROC) analysis demonstrated that the risk model exhibited favorable prognostic accuracy, with area under the curve (AUC) values of 0.871, 0.855, and 0.841 for 1, 3, and 5-year overall survival, respectively (**Figure 6A**). In addition, calibration curve analysis was performed to evaluate the agreement between the model-predicted and observed survival probabilities. The calibration curves showed generally acceptable consistency between predicted and observed overall survival probabilities, particularly for 3- and 5-year OS (**Supplementary Figure 3A**). Decision curve analysis was further conducted to assess the potential clinical utility of the prognostic model for survival risk stratification. Compared with the hypothetical all-positive and none-positive reference strategies, the prognostic model provided a higher net benefit across a range of threshold probabilities, supporting its potential value for identifying high-risk patients (**Supplementary Figure 3B**). Kaplan–Meier survival analysis showed that patients in the high-risk group had significantly shorter overall survival compared with those in the low-risk group (p < 0.0001; **Figure 6B**). Risk score distribution analysis revealed that glioblastoma samples exhibited significantly higher risk scores compared with lower-grade glioma and normal brain tissues (**Figure 6C**). In addition, risk scores increased progressively with advancing WHO grade, with grade IV tumors showing the highest scores (**Figure 6D**). Stratification by molecular features demonstrated that patients with unmethylated MGMT promoter status exhibited significantly higher risk scores than those with methylated MGMT promoters (**Figure 6E**). Moreover, patients with IDH wild-type tumors displayed higher risk scores compared with those harboring IDH mutations (**Figure 6F**). Additionally, similar results were obtained from the CGGA cohort, where ROC analysis (**Figure 6G**) and Kaplan-Meier survival analysis (**Figure 6H**) further confirmed the model’s predictive value in an independent dataset.

### 3.5 Expression pattern and clinical relevance of KLF10 in glioma

**Figure 7:**
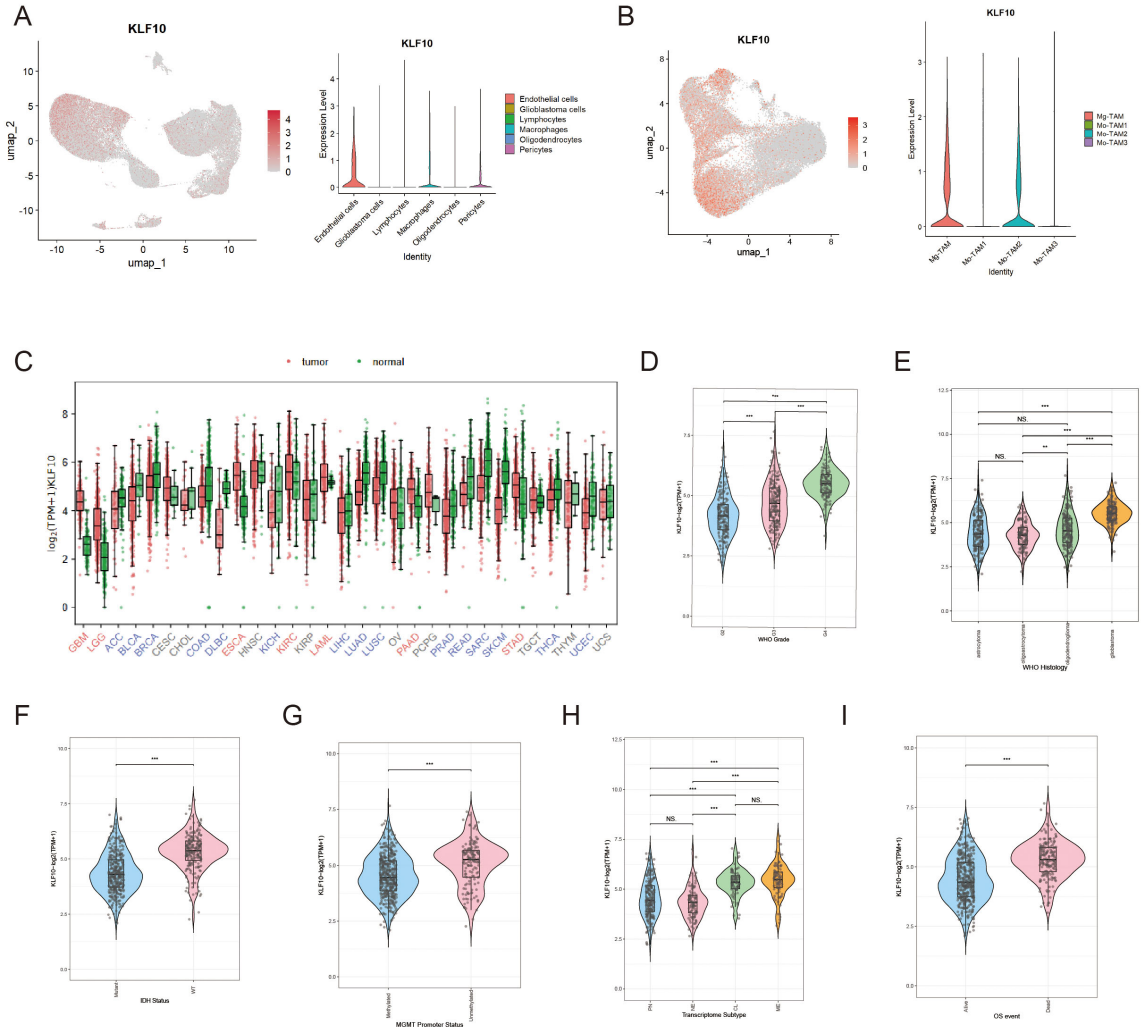
Expression pattern and clinical relevance of KLF10 in glioma **(A)** UMAP visualization showing the distribution of KLF10 expression across major cell types within the glioblastoma microenvironment, with relatively higher expression in macrophages, endothelial cells, and pericytes. **(B)** Violin plot illustrating KLF10 expression across myeloid subpopulations, showing elevated expression in tumor-associated macrophages (TAMs), particularly in the Mo-TAM2 subset. **(C)** Pan-cancer analysis comparing KLF10 expression between tumor and corresponding normal tissues across multiple cancer types. **(D)** KLF10 expression across different WHO grades of glioma, showing a progressive increase with advancing grade. **(E)** Comparison of KLF10 expression among glioblastoma, astrocytoma, and oligodendroglioma. **(F)** Differential KLF10 expression between IDH wild-type and IDH-mutant gliomas. **(G)** Comparison of KLF10 expression according to MGMT promoter methylation status. **(H)** KLF10 expression across glioblastoma transcriptomic subtypes, showing the highest expression in the mesenchymal (MES) subtype. **(I)** Comparison of KLF10 expression between patients with and without disease progression or death. NS, not significant; **P < 0.01; ***P < 0.001.

Among the five candidate genes, KLF10, GPR84, and ZFAND2A were identified as risk-associated factors. Because GPR84 and ZFAND2A have already been implicated in macrophage-related tumor biology (12–14), whereas KLF10 remains largely unexplored in GBM-associated TAMs, we selected KLF10 for further analysis. The expression pattern and clinical relevance of KLF10 were further investigated. UMAP visualization showed that KLF10 was broadly expressed across multiple cell types in the glioblastoma microenvironment, with relatively higher expression in macrophages, endothelial cells, and pericytes compared with other cell populations (**Figure 7A**). Further stratification of myeloid subsets revealed that KLF10 expression was elevated in tumor-associated macrophages, particularly in the Mo-TAM2 subset, compared with other Mo-TAM subpopulations (**Figure 7B**).

Pan-cancer analysis demonstrated that KLF10 expression exhibited marked heterogeneity across different cancer types compared with normal tissues (**Figure 7C**). In glioma samples, KLF10 expression increased progressively with advancing WHO grade, with the highest expression observed in grade IV tumors (**Figure 7D**). Consistently, KLF10 expression was significantly higher in glioblastoma compared with astrocytoma and oligodendroglioma (**Figure 7E**).

Stratified analysis based on molecular features showed that KLF10 expression was significantly higher in IDH wild-type tumors than in IDH-mutant tumors (**Figure 7F**), and was also elevated in tumors with an unmethylated MGMT promoter compared with those with MGMT promoter methylation (**Figure 7G**). Moreover, analysis according to transcriptomic subtypes revealed that KLF10 expression was higher in the mesenchymal (MES) and classical (CL) subtypes compared to the neural (NE) and proneural (PN) subtypes (**Figure 7H**). KLF10 expression was higher in patients with death events than in those without death events (**Figure 7I**).

**Figure 8:**
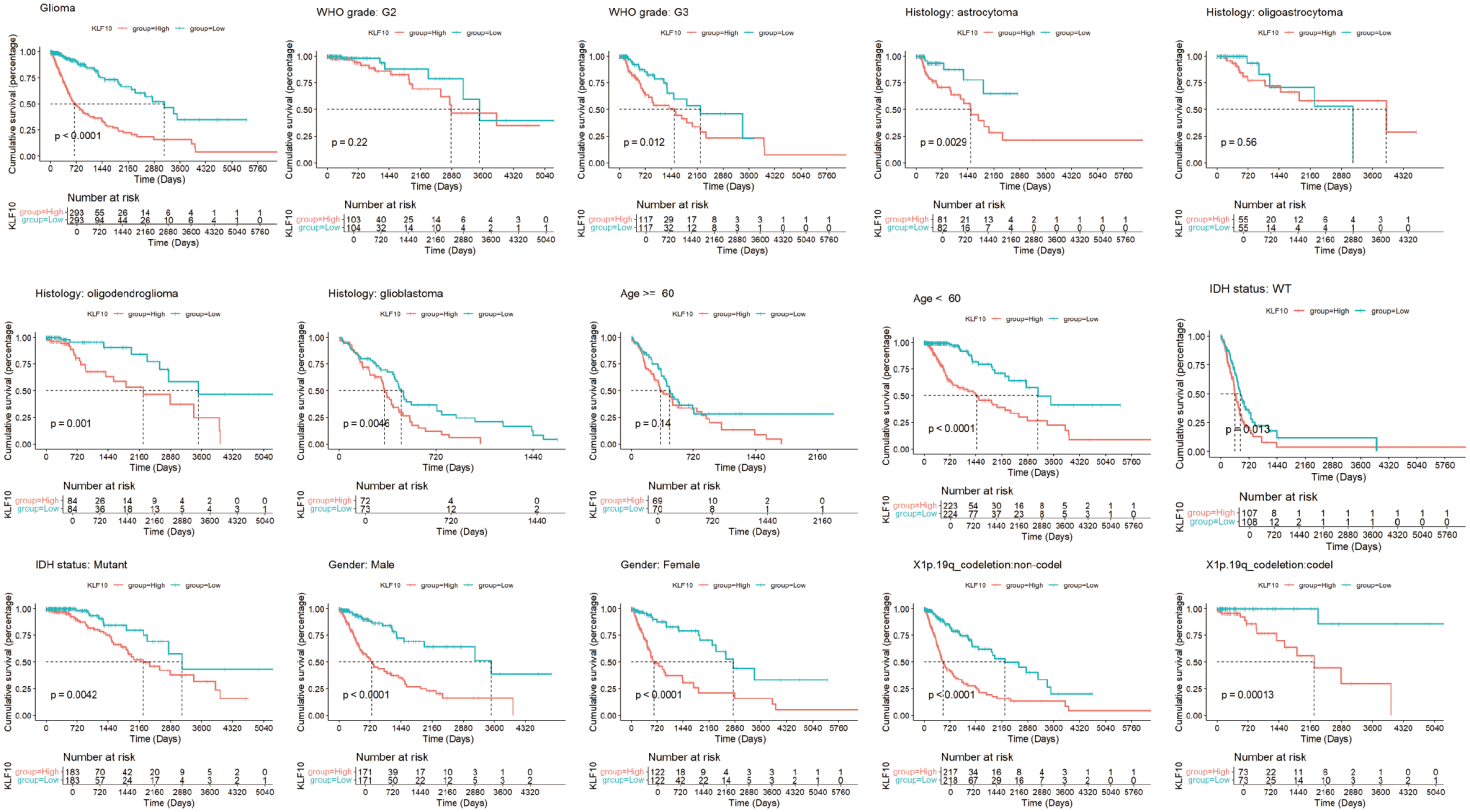
Association between KLF10 expression and survival outcomes in glioma. Kaplan–Meier survival analyses were performed to evaluate the prognostic significance of KLF10 expression.

Kaplan-Meier survival analysis revealed that higher KLF10 expression was associated with poorer overall survival in glioma patients (p < 0.0001, **Figure 8**). This correlation was significant across different WHO grades (G3, p = 0.012; G2, p < 0.0001), histological subtypes (astrocytoma, p = 0.0029; oligodendroglioma, p = 0.001), and in patients younger than 60 (p < 0.0001). KLF10 expression was also linked to survival in IDH wild-type tumors (p = 0.013) and genetic alterations such as 1p/19q non-deletion (p < 0.0001) and codeletion (p = 0.00013).

### 3.6 KLF10 expression correlates with glioblastoma progression and prognosis

**Figure 9:**
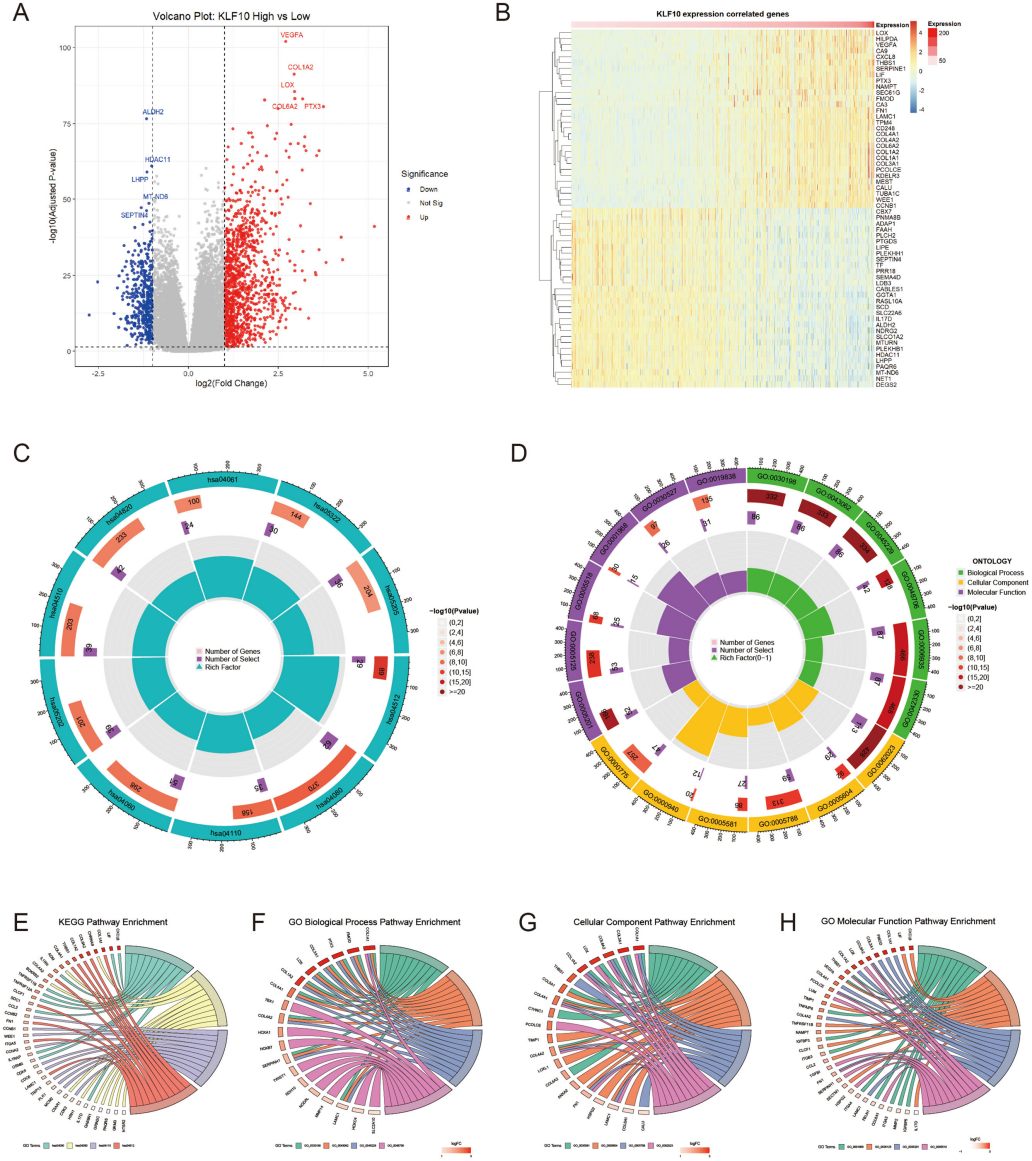
KLF10-associated transcriptional programs and functional enrichment analysis in glioblastoma **(A)** Volcano plot showing differentially expressed genes between the KLF10-high and KLF10-low groups. **(B)** Heatmap illustrating the expression patterns of KLF10-correlated genes, highlighting distinct gene clusters co-expressed with KLF10. **(C)** KEGG pathway enrichment analysis of KLF10-associated genes. **(D)** GO pathway enrichment analysis of KLF10-associated genes. **(E)** The chord diagram of representative KEGG pathways enriched in KLF10-related gene sets. **(F–H)** The chord diagram illustrated the associated pathways and genes of KLF10-related genes in the enrichment of GO biological processes, cellular components, and molecular functions.

The differential expression of genes associated with KLF10 was analyzed using a volcano plot, which showed significant upregulation and downregulation of several genes in the KLF10 high expression group compared to the KLF10 low group (**Figure 9A**). Notable upregulated genes in the KLF10 high group included VEGFA, COL1A2, LOX, COL6A2, and PTX3, while genes with downregulated expression included ALDH2, HDAC11, LHPP, MT-ND6, and SEPTIN4. The heatmap (**Figure 9B**) further illustrated the expression profiles of KLF10-correlated genes, highlighting distinct clusters of genes that were co-expressed with KLF10 in glioblastoma samples.

To investigate the functional implications of KLF10 expression, we conducted KEGG pathway enrichment analysis (**Figure 9C**; **Table 1**) and GO pathway enrichment analysis (**Figure 9D**; **Table 2**). Notably, KLF10-associated genes were enriched in pathways related to extracellular matrix organization, cytokine activity, and chemotaxis, suggesting that KLF10 may be associated with immune responses and cell invasion-related processes in glioblastoma. Further KEGG pathway enrichment analysis indicated that KLF10-related genes were involved in processes such as ECM-receptor interaction, cell cycle regulation, and cytokine-cytokine receptor interaction, among others (**Figure 9E**). These findings suggested that KLF10 may be associated with tumor progression-related and immune microenvironment-related transcriptional programs in glioblastoma.

**Table 1:** Supplementary information of KEGG analysis.

ID	Description	GeneRatio	BgRatio	p.adjust	Count
hsa04512	ECM-receptor interaction	29/733	89/9522	2.51E-09	29
hsa04080	Neuroactive ligand-receptor interaction	62/733	370/9522	4.68E-07	62
hsa04110	Cell cycle	35/733	158/9522	8.32E-07	35
hsa04060	Cytokine-cytokine receptor interaction	51/733	298/9522	3.12E-06	51
hsa05202	Transcriptional misregulation in cancer	39/733	201/9522	3.46E-06	39
hsa04510	Focal adhesion	39/733	203/9522	3.81E-06	39
hsa04820	Cytoskeleton in muscle cells	42/733	233/9522	6.69E-06	42
hsa04061	Viral protein interaction with cytokine and cytokine receptor	24/733	100/9522	1.37E-05	24
hsa05322	Systemic lupus erythematosus	30/733	144/9522	1.37E-05	30
hsa04518	Integrin signaling	31/733	154/9522	1.75E-05	31

**Table 2:** Supplementary information of GO analysis.

Ontology	ID	Description	GeneRatio	BgRatio	p.adjust	Count
BP	GO:0030198	extracellular matrix organization	86/1384	332/18888	3.12E-22	86
BP	GO:0043062	extracellular structure organization	86/1384	333/18888	3.12E-22	86
BP	GO:0045229	external encapsulating structure organization	86/1384	334/18888	3.12E-22	86
BP	GO:0048706	embryonic skeletal system development	42/1384	128/18888	5.26E-14	42
BP	GO:0006935	chemotaxis	87/1384	466/18888	5.09E-13	87
BP	GO:0042330	taxis	87/1384	468/18888	5.55E-13	87
CC	GO:0062023	collagen-containing extracellular matrix	113/1455	428/19894	1.26E-31	113
CC	GO:0005604	basement membrane	29/1455	92/19894	2.77E-09	29
CC	GO:0005788	endoplasmic reticulum lumen	59/1455	313/19894	3.63E-09	59
CC	GO:0005581	collagen trimer	27/1455	86/19894	8.15E-09	27
CC	GO:0000940	outer kinetochore	12/1455	20/19894	2.65E-07	12
CC	GO:0000775	chromosome, centromeric region	47/1455	257/19894	6.02E-07	47
MF	GO:0005201	extracellular matrix structural constituent	52/1428	166/18522	6.69E-16	52
MF	GO:0005125	cytokine activity	53/1428	238/18522	1.03E-09	53
MF	GO:0005518	collagen binding	25/1428	68/18522	6.63E-09	25
MF	GO:0001968	fibronectin binding	15/1428	30/18522	3.79E-07	15
MF	GO:0030527	structural constituent of chromatin	26/1428	97/18522	3.85E-06	26
MF	GO:0019838	growth factor binding	31/1428	135/18522	7.27E-06	31

GO Biological Process analysis showed enrichment in extracellular matrix organization, extracellular structure organization, and external encapsulating structure organization, among others (**Figure 9F**). GO Cellular Component analysis revealed that KLF10-correlated genes were predominantly enriched in structures such as the basement membrane, collagen-containing extracellular matrix, and cell-substrate junctions, among others (**Figure 9G**). Finally, GO Molecular Function analysis showed significant enrichment in genes involved in cytokine activity, extracellular matrix structural constituent, and growth factor binding, among others (**Figure 9H**), suggesting that KLF10-associated genes may be involved in cellular communication and extracellular signaling processes related to glioblastoma progression.

### 3.7 Glioblastoma cells upregulated KLF10 expression in macrophages

**Figure 10:**
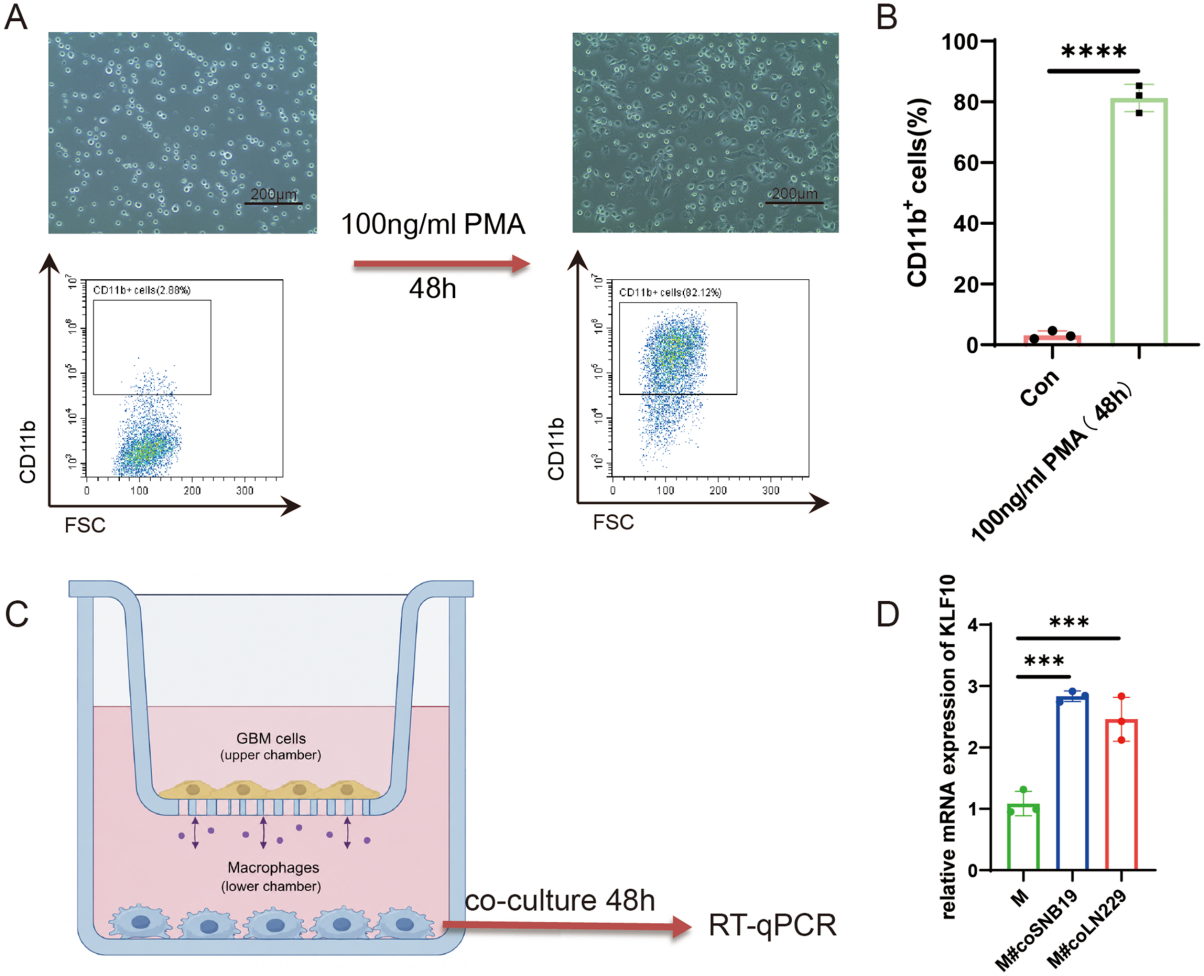
Glioblastoma cells upregulated KLF10 expression in macrophages **(A)** Schematic illustration of THP-1 monocyte differentiation into macrophages induced by PMA treatment (100 ng/mL for 48 h). **(B)** Flow cytometric analysis confirming successful macrophage differentiation, as evidenced by a significant increase in CD11b^+^ cells after PMA induction. **(C)** Schematic diagram of the co-culture system established between glioblastoma cells and macrophages. **(D)** RT-qPCR analysis showing that co-culture with glioblastoma cells significantly upregulated KLF10 expression in macrophages. Data are presented as mean ± SD from three independent experiments. Statistical significance was assessed by two-tailed Student’s t-test or one-way ANOVA with Tukey’s *post hoc* test. ns, not significant; ***P < 0.001; ****P < 0.0001.

THP-1 cells were treated with 100 ng/ml PMA for 48 hours to induce macrophage differentiation (**Figure 10A**). The expression of the macrophage marker CD11b^+^ was subsequently verified by flow cytometry. A significant increase in CD11b^+^ cells was observed following induction (**Figure 10B**). To investigate whether glioblastoma affects KLF10 expression in macrophages, a co-culture system of glioblastoma cells and macrophages was established (**Figure 10C**). RT-qPCR analysis indicated that co-culture with glioblastoma cells significantly upregulated KLF10 expression in macrophages (**Figure 10D**).

### 3.8 KLF10-overexpressing macrophages were associated with increased proliferation, migration, and invasion of glioblastoma cells *in vitro*

**Figure 11:**
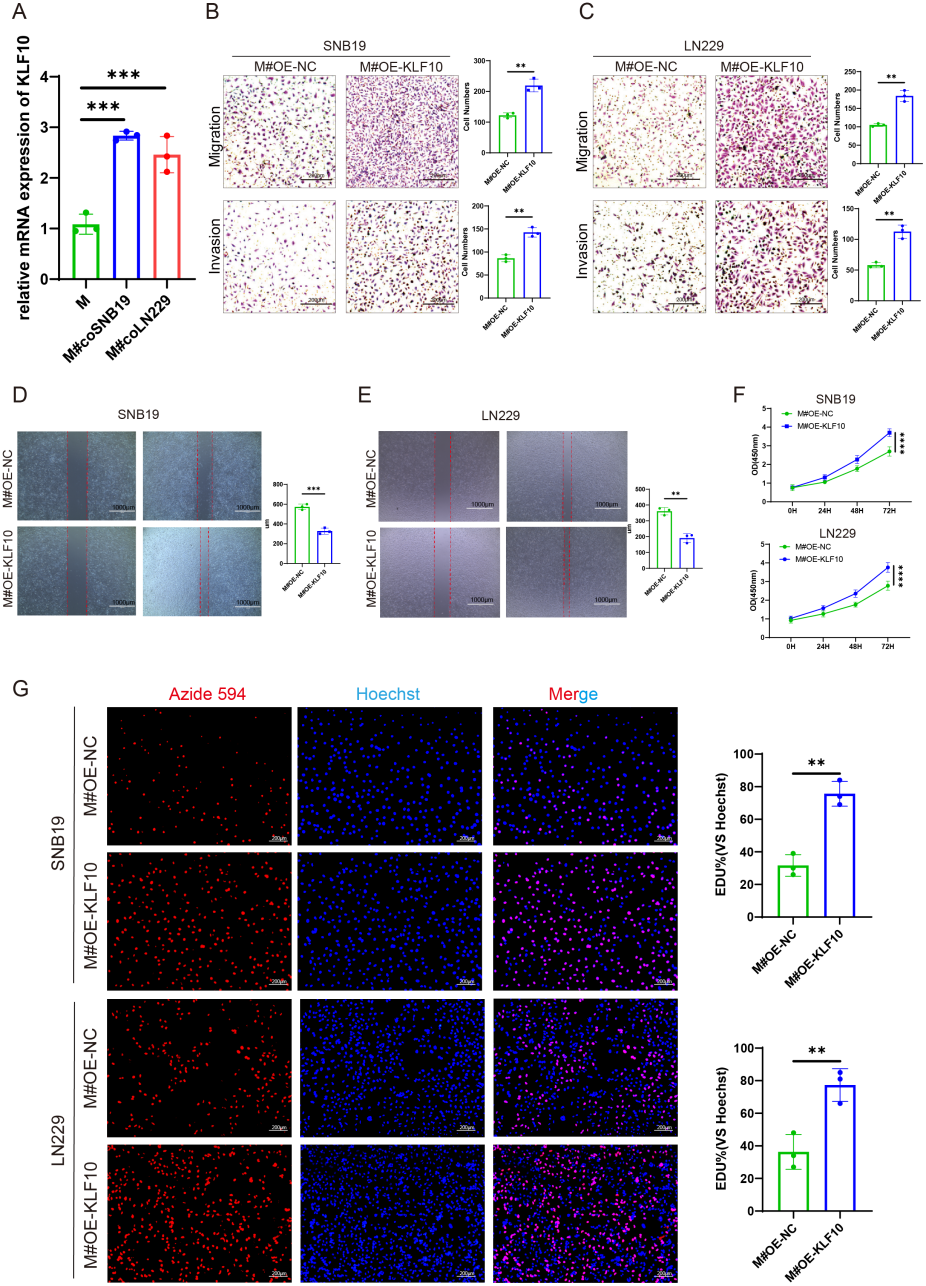
KLF10-overexpressing macrophages were associated with increased proliferation, migration, and invasion of glioblastoma cells *in vitro* **(A)** Validation of KLF10 overexpression in macrophages following plasmid transfection, as determined by RT-qPCR. **(B, C)** Transwell migration and invasion assays showing that KLF10-overexpressing macrophages were associated with increased migratory and invasive capacities of glioblastoma cells. **(D, E)** Wound-healing assays showing increased migration of glioblastoma cells co-cultured with KLF10-overexpressing macrophages. **(F, G)** CCK-8 and EdU incorporation assays showing that co-culture with KLF10-overexpressing macrophages was associated with increased glioblastoma cell proliferation. Data are presented as mean ± SD from three independent experiments. Statistical significance was assessed by two-tailed Student’s t-test or one-way ANOVA with Tukey’s *post hoc* test. ns, not significant; **P < 0.01; ***P < 0.001; ****P < 0.0001.

To investigate how macrophages with high KLF10 expression affect glioblastoma cell progression, macrophages overexpressing KLF10 were generated through plasmid transfection. The overexpression efficiency was confirmed by RT-qPCR (**Figure 11A**). A co-culture system of macrophages overexpressing KLF10 and glioblastoma cells was subsequently established. The effects of KLF10-overexpressing macrophages on glioblastoma cell proliferation, migration, and invasion capacities were evaluated using Transwell migration and invasion assays, cell scratch assays, CCK-8 assays, and EdU incorporation assays. Transwell migration and invasion assays demonstrated that KLF10-overexpressing macrophages were associated with increased migration and invasion capacities of glioblastoma cells (**Figures 11B, C**). Cell scratch assays revealed that co-culture with macrophages overexpressing KLF10 was accompanied by increased migratory capacity of glioblastoma cells (**Figures 11D, E**). CCK-8 cell proliferation assays and EdU incorporation assays indicated that co-culture with KLF10-overexpressing macrophages was associated with increased proliferative capacity of glioblastoma cells (**Figures 11F, G**).

## 4 Discussion

The TME of glioblastoma was complex, consisting of various types of tissue-resident cells and infiltrating non-tumor cells, with distinct spatial characteristics, which together formed a unique glioblastoma structure (15, 16). In glioblastoma, TAMs were one of the most important immune cell types in the tumor microenvironment, playing a key role in tumor progression, immune evasion, and treatment resistance (17). Recent research has highlighted the potential for TAMs to play crucial and specific roles in glioblastoma recurrence. Glioblastoma recurrence has been associated with an increased abundance of immune cell components and the presence of distinct myeloid cell states within the tumor microenvironment (18). Previous studies demonstrated a significant increase in tumor-associated macrophage infiltration in mesenchymal-like recurrent glioblastoma, which was inversely correlated with tumor purity. These observations supported a critical role for TAMs in favoring mesenchymal transition and promoting GBM progression at recurrence (19). Following anti-angiogenic therapy, recurrent glioblastoma was characterized by enhanced infiltration of myeloid cells in both the tumor core and infiltrative areas, and elevated CD11b^+^ cell abundance was significantly correlated with reduced overall survival (6). However, the key molecules that regulated the functional changes of TAMs in recurrent glioblastoma were not fully elucidated.

To further investigate the potential key molecules that regulate the functional changes of TAMs in recurrent glioblastoma, we identified significant heterogeneity among macrophage subsets, particularly within the Mo-TAM2 subset, through scRNA-seq analysis. The proportion of this subset was significantly increased in recurrent glioblastoma and may be closely associated with poor prognosis in glioblastoma. Further investigation revealed that KLF10 was identified as a potential marker for this subset of recurrence-associated macrophages and was found to be correlated with glioblastoma prognosis and clinical features. The enrichment analyses in this study suggested that KLF10-associated genes were linked to extracellular matrix remodeling, cytokine signaling, and tumor-progression-related programs. However, these enriched pathways should be interpreted as predicted transcriptional associations rather than experimentally validated downstream mechanisms of KLF10, and future pathway validation experiments are required to establish their biological relevance.

The expression and function of KLFs were altered in human cancers, and they regulated cancer cell proliferation and apoptosis, metastasis, the tumor microenvironment, and cancer stem cells (8). KLF10 was found to be downregulated in multiple myeloma tissues, where it promoted apoptosis and inhibited the cell cycle (20). In pancreatic ductal adenocarcinoma, KLF10 expression was downregulated, leading to enhanced tumor invasiveness and increased tumor stemness (21). In contrast to these studies, KLF10 expression was significantly elevated in glioblastoma and was associated with poor prognosis. *In vitro*, glioblastoma cells upregulated KLF10 expression in THP-1-derived macrophages, and macrophages with ectopic KLF10 overexpression were associated with increased proliferation, migration, and invasion of glioblastoma cells. These findings suggest that KLF10 may be related to a GBM-supportive macrophage state. The current data support an association between KLF10 expression and recurrence-related GBM-supportive TAM features. However, further experimental validation is required to determine its functional and clinical significance. In addition, macrophage polarization was not experimentally evaluated using canonical markers or cytokine profiling. Therefore, the observed phenotype should be interpreted as a GBM-supportive macrophage state rather than a classical M1/M2 polarization state.

The biological significance of KLF10 in GBM progression remains to be validated in patient-derived and orthotopic glioblastoma models. Moreover, because ectopic KLF10 overexpression may not fully reflect physiological conditions, further experiments are needed to clarify the role of endogenous KLF10. In addition, THP-1-derived macrophages cannot fully recapitulate primary human macrophages or patient-derived TAMs, and further validation in these models is required. Finally, since KLF10 was identified mainly through transcriptomic datasets, its clinical value as a prognostic biomarker requires further protein-level and spatial validation in independent clinical cohorts.

## 5 Conclusion

In conclusion, TAMs in both primary and recurrent glioblastoma immune microenvironments exhibited heterogeneity, and KLF10 may be involved in recurrence-associated macrophage features in GBM. Glioblastoma cells upregulated KLF10 expression in THP-1-derived macrophages, and KLF10-overexpressing macrophages were associated with increased GBM-cell proliferation, migration, and invasion *in vitro*. These findings suggest that KLF10 may serve as a candidate molecule associated with recurrence-related TAM features and macrophage–GBM interactions. However, THP-1-derived macrophages cannot fully recapitulate the biological complexity of primary human macrophages or patient-derived TAMs, and further validation in clinically relevant macrophage models is required.

## Data Availability

The original contributions presented in the study are included in the article/**Supplementary Material**. Further inquiries can be directed to the corresponding authors.
