# Supplementary material for: Single-cell analysis reveals KLF10-high macrophages induced by glioblastoma cells are associated with tumor progression
Source: Front Immunol. 2026 Jul 20;17:1820802. doi: 10.3389/fimmu.2026.1820802 (PMC13429638; doi:10.3389/fimmu.2026.1820802)
Supplement: Supplementary file 2 [file Table1.docx]

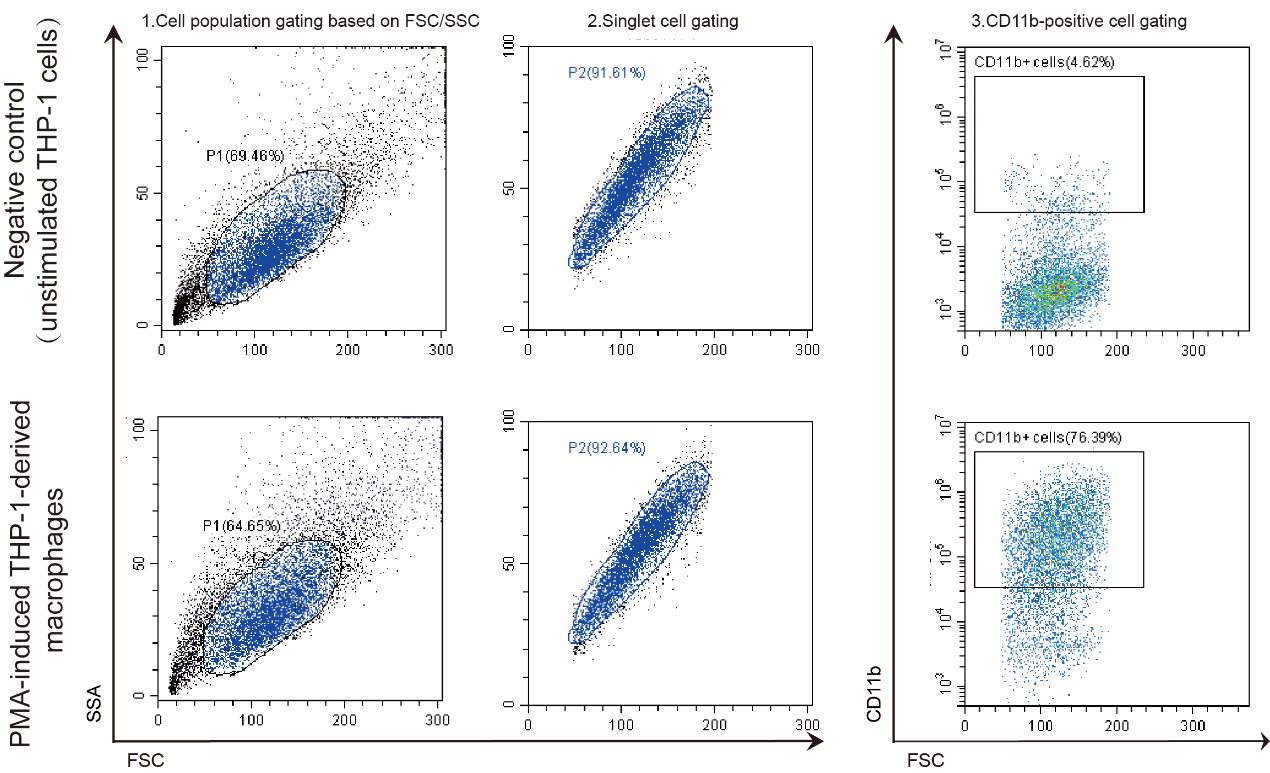
 Figure S1. Flow cytometry gating strategy for CD11b detection. The main cell population was first selected based on FSC/SSC profiles, followed by singlet cell gating. Unstimulated THP-1 cells were used as the negative control to define the CD11b-positive gate, and the CD11b expression in PMA-induced THP-1-derived macrophages was analyzed.
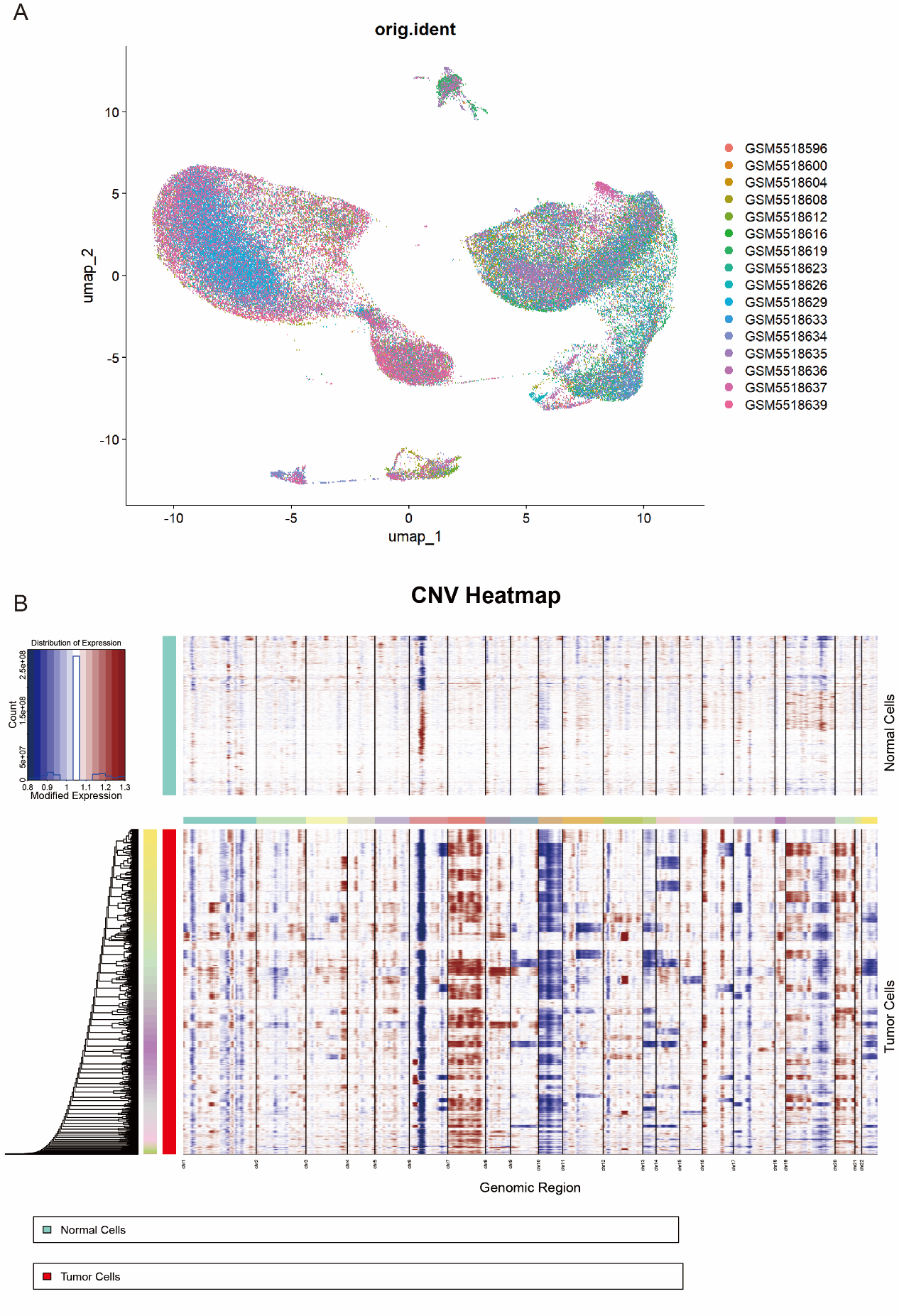
Figure S2. (A)UMAP visualization of scRNA-seq data colored by original sample identity after batch-effect correction. (B)CNV inference analysis of single-cell RNA-seq data using inferCNV. Non-malignant cell populations were used as reference cells. Compared with normal/reference cells, GBM cells showed broad chromosomal copy number alterations, supporting the neoplastic identity of the annotated GBM cell population.


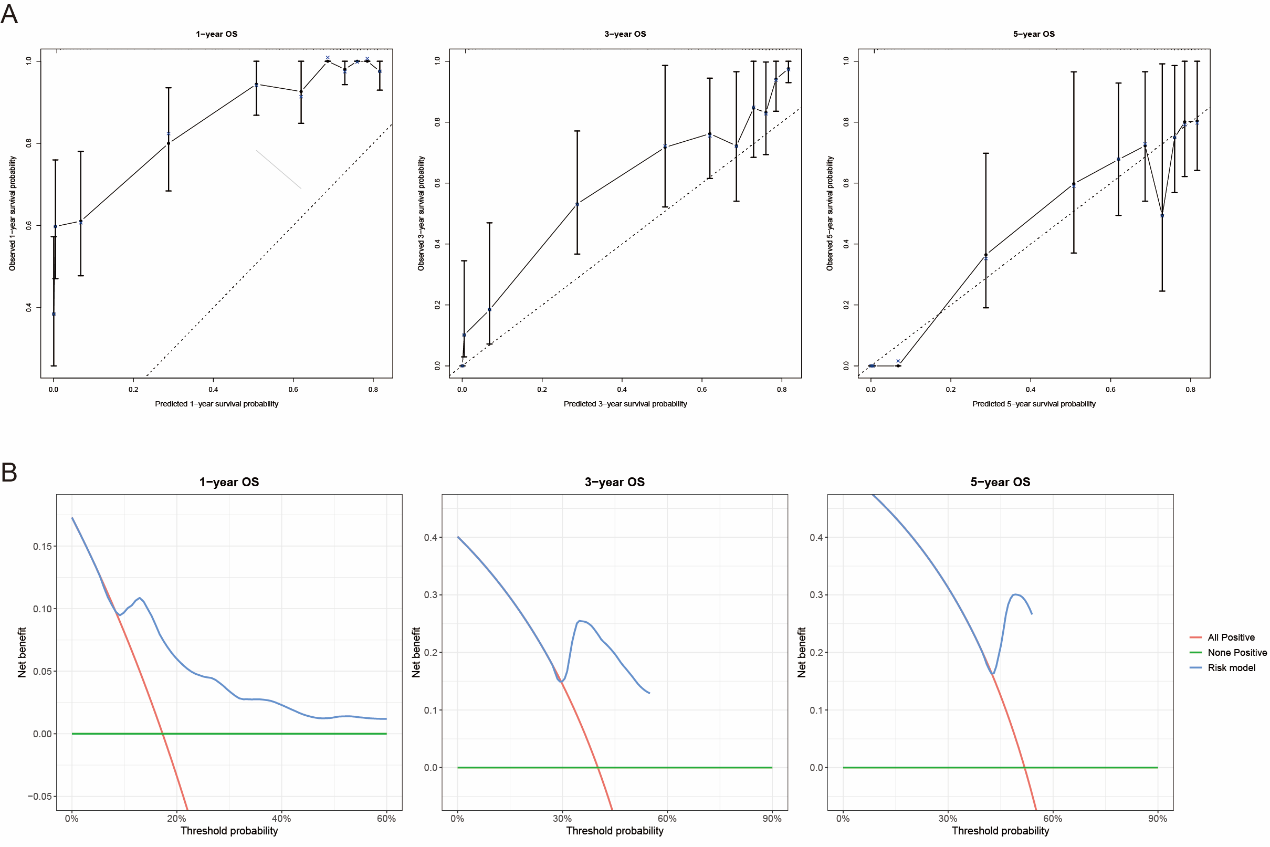


Figure S3. Calibration curve analysis and decision curve analysis of the prognostic model. (A) Calibration curves for predicting 1-, 3-, and 5-year overall survival. The dashed line represents the ideal prediction, and the solid line represents the performance of the prognostic model. (B) Decision curve analysis for 1-, 3-, and 5-year overall survival.
